# ECMO in hinterland—feasibility and challenges (case-based narrative review)

**DOI:** 10.3389/fmed.2026.1778714

**Published:** 2026-09-28

**Authors:** Vamshi Krishna Jonnalagedda, Kollengode Ramanathan, Pranay L. Oza, Arpan Chakraborty, Poonam Malhotra, Nagarajan Ganapathy, Rakesh Venuturumilli, Dipanjan Chatterjee, Ahsina Jahan, Rajesh C. Mishra

**Affiliations:** 1 Department of Critical Care, KIMS Hospitals Secunderabad, Hyderabad, India; 2 Department of Cardiothoracic Intensive Care, National University Hospital (NUH), Singapore, Singapore; 3 Department of ECMO & Cardiology, RVMH, Mumbai, India; 4 Department of Critical Care and ECMO Services, Apollo Multispeciality Hospital, Kolkata, India; 5 Department of Anaesthesia and Critical Care, AIIMS, New Delhi, India; 6 Critical Care Unit, Dhanvantri Institute of Medical Education and Research, Erode, India; 7 Department of Critical Care & ECMO, Fortis Gleneagles Hospitals, Hyderabad, India; 8 Department of Critical Care and ECMO Services, Manipal Hospital EM Bypass, Kolkata, India; 9 Department of ICU and Emergency, ICU Specialized and General Hospital, Dhaka, Bangladesh; 10 Department of Critical Care Medicine, Sterling Hospitals Gurukul, Ahmedabad, India

**Keywords:** ethics, extra corporeal membrane oxygenation, hub and spoke model, out reach program, periphral centre, tele ECMO, transfer protocol

## Abstract

Extracorporeal membrane oxygenation (ECMO) is one of the most advanced forms of cardiopulmonary support used for severe respiratory and/or cardiac failure when other conventional treatments are not optimal. However, the practice or use of ECMO remains predominant in tertiary centres due to its resource-intensive nature (human, skill, technical, and financial), thereby limiting access in peripheral and resource-constrained regions. So, this narrative review examines the feasibility and challenges of establishing ECMO services in such settings (described as the hinterland) through three major models: outreach ECMO programmes, hub-and-spoke systems, and tele-ECMO networks. Previous reviews have discussed ECMO organisation, transport, and regionalisation in general. Few have addressed the practical question of which ECMO model a hinterland hospital should choose and when. This review aims to fill that gap by directly comparing three practical models. Using case-based discussions and available literature, the review outlines the common foundational requirements for safe ECMO delivery, including infrastructure, logistics, multidisciplinary teamwork, transport preparedness, ethical governance, and protocol standardisation. It explores the advantages and disadvantages of each model, with a focus on components such as early referral, expert oversight, safe interfacility transfer, and a viable long-term partnership between peripheral and expert ECMO centres to improve patient outcomes. The review discusses ethical and financial aspects of ECMO program. It specifically explores the future role of telemedicine, artificial intelligence, and digital connectivity in expanding ECMO across all areas. To conclude, the review demonstrates that, with proposed models of structured systems and appropriate training, ECMO can be safely extended to unexplored regions while maintaining quality and patient safety.

## Introduction

The expanding use of extracorporeal membrane oxygenation (ECMO) has transformed modern critical care, yet its availability remains uneven, particularly in peripheral or resource-limited centres (1). Initiating ECMO in peripheral or resource-limited environments is often perceived as challenging, not only because of the technical complexity but also due to the systems-level demands that accompany such high-acuity care. Updated 2021 ELSO guidelines on ECMO for COVID-19 address how to plan ECMO in areas with limited experience (2–4). Beyond theoretical concerns, practical issues can be substantial. Peripheral centres must contend with constrained resources, unfamiliar environments, limited staff exposure, and logistical barriers that are rarely encountered in major ECMO hubs. ELSO and other organisations provide general guidance on ECMO organisation, but there is limited guidance on a practical issue faced by hinterland hospitals: choosing among outreach, hub-and-spoke, or tele-ECMO models. This review addresses this gap by directly comparing the three approaches.

All such peripheral centres are referred to as “hinterland” institutions in this article; For an international audience, terms such as resource-limited, non-tertiary, spoke, and satellite centres are commonly used in the LMIC literature and broadly refer to similar settings. In this review, a hinterland centre is defined not simply by its distance from a major city but by its ability to independently support an ECMO programme. This depends on ICU capacity, experience with advanced organ support, availability of trained intensivists, perfusionists and ICU nurses, and the strength of the referral network. Consequently, even a tertiary hospital in a metropolitan setting may fall within this definition if it lacks these capabilities.

This article examines feasible strategies for establishing safe ECMO initiation in resource-limited or peripheral institutions, outlines the challenges likely to be encountered, and proposes mitigation frameworks informed by relevant case-based discussions.

## Methods

This narrative review was developed to evaluate the feasibility, challenges, and organisational models for establishing ECMO services in hinterland centres, i.e., resource-constrained settings. We searched for peer-reviewed articles using keywords extracorporeal membrane oxygenation, outreach programme, hub-and-spoke model, tele-ECMO, transfer protocol, ethics, and peripheral centre. We searched over a 25-year period, from Jan 2001 to March 2026. We searched the PubMed database to find the relevant articles. These terms were combined using Boolean operators, for example: (“extracorporeal membrane oxygenation” OR “ECMO”) AND (“outreach” OR “hub-and-spoke” OR “tele-ECMO” OR “transfer” OR “ethics” OR “peripheral centre” OR “regionalisation”). Two authors independently screened the titles and abstracts. Any disagreements were resolved through discussion, after which the full texts of potentially eligible articles were assessed against the stated inclusion and exclusion criteria.

Eligible publications comprised ELSO guidelines, consensus statements, position papers, original research, systematic reviews, and case reports pertinent to ECMO delivery outside high-volume tertiary centres. Articles addressing purely technical aspects of ECMO, without bearing on programme development, transport, networking, or peripheral implementation, were excluded, as were those dealing with ECPR and transport in this context, as well as non-English publications lacking an accessible translation. Titles and abstracts were first screened for relevance, following which the corresponding texts were reviewed. Of the 114 articles initially identified, 64 key references were ultimately selected for inclusion.

Due to the significant heterogeneity in study designs and international healthcare systems, the findings were synthesised narratively to illustrate practical operational challenges and potential solutions.

As this is a narrative review, a PRISMA flow diagram was not used. Instead, reporting was guided by the SANRA framework (Scale for the Assessment of Narrative Review Articles) (5), which emphasises a transparent search strategy, appropriate referencing, and description of the level of evidence rather than a systematic-review-style screening diagram. We recognise that restricting the search to PubMed is a limitation, which is discussed further in the Critical Analysis section.

### Need for developing ECMO programme in hinterland

ELSO has been a strong proponent of regional network development and has published guidelines for Extracorporeal Life Support (ECLS) centres addressing patient selection and training for newly established teams (6, 7). The ESOI complements these efforts by offering continuing education and hands-on training programmes aimed at building competency and expanding ECMO expertise across centres (8).

Peripheral hospitals nonetheless face considerable challenges, particularly in delivering subspecialty services that demand specialised personnel and equipment. Patients and families are often reluctant to place their trust in unfamiliar medical teams in critical situations, especially when the recommended therapy is high-end, uncertain in benefit, and costly. Limited awareness of such advanced therapies further complicates obtaining timely, informed consent for appropriate treatment. Notably, nearly 70% of India's population lives in resource-limited settings—Tier II and Tier III cities—where healthcare infrastructure more closely resembles that of low- and middle-income countries than of major metropolitan centres. Transferring patients with severe cardiogenic shock (secondary to aluminium phosphide poisoning or acute coronary events) or severe ARDS with type II respiratory failure to a tertiary centre is fraught with difficulty in the absence of ECMO support (4, 9). The financial burden of such transfers is compounded by the dual costs of private healthcare and relocation to an urban centre. Beyond these material costs, families also contend with the psychological and logistical toll of an unpredictable clinical course in a critically ill relative, often while displaced indefinitely in unfamiliar surroundings.

To establish ECMO services in regional and remote settings, the literature identifies three primary delivery models: the Outreach ECMO programme, the Hub-and-Spoke framework, and the Tele-ECMO platform. Each paradigm offers distinct clinical and logistical advantages while presenting unique operational constraints (10–14). In subsequent sections, the authors first elaborated on common considerations, then on the specifics of each model. Table 1 compares the three models. Figures 1–3 show the flow charts of the different models.

**Figure 1:**
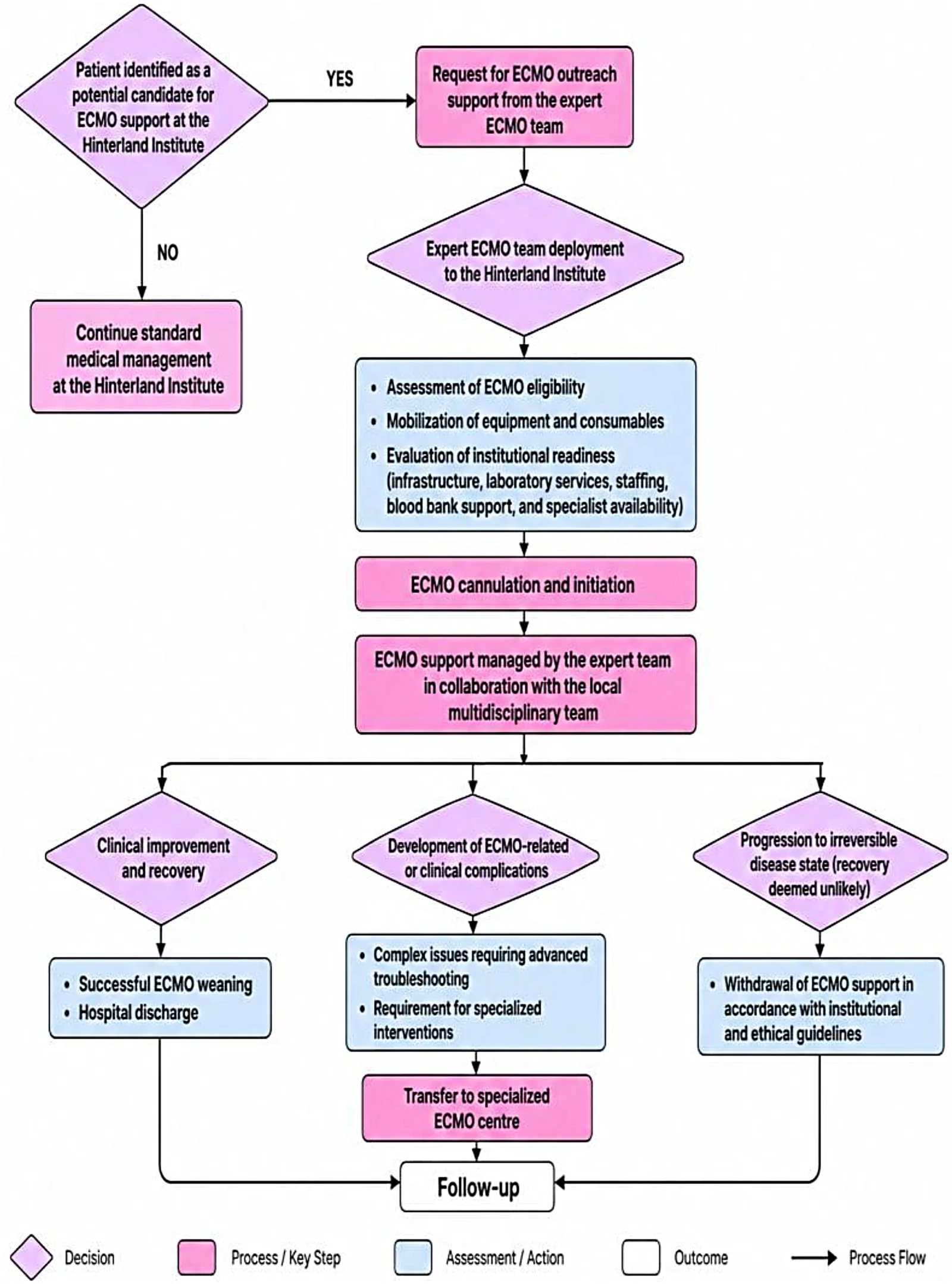
ECMO outreach programme flowchart. ECMO, extracorporeal membrane oxygenation.

**Figure 2:**
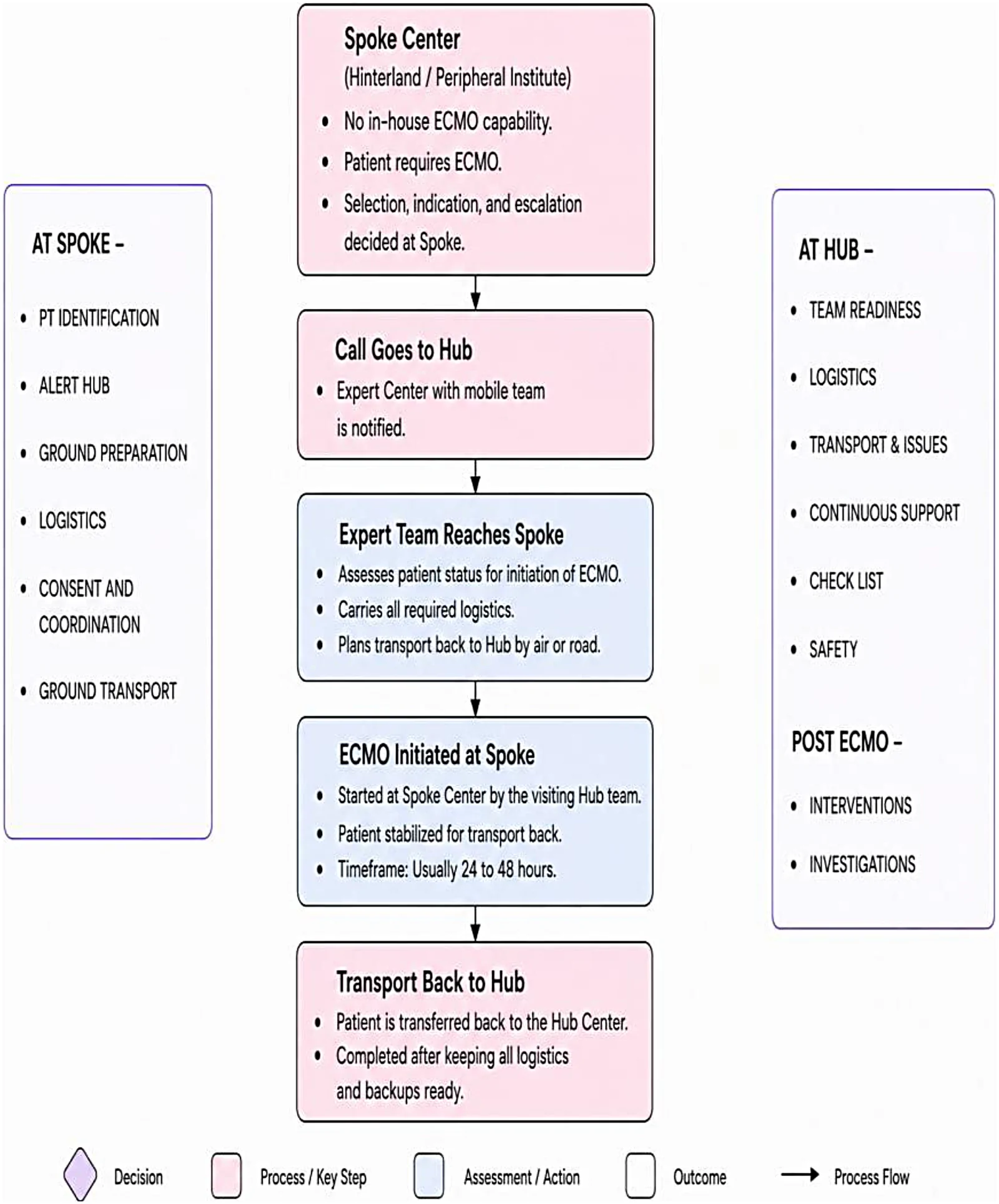
Hub-And-Spoke model flow diagram. ECMO, extracorporeal membrane oxygenation.

**Figure 3:**
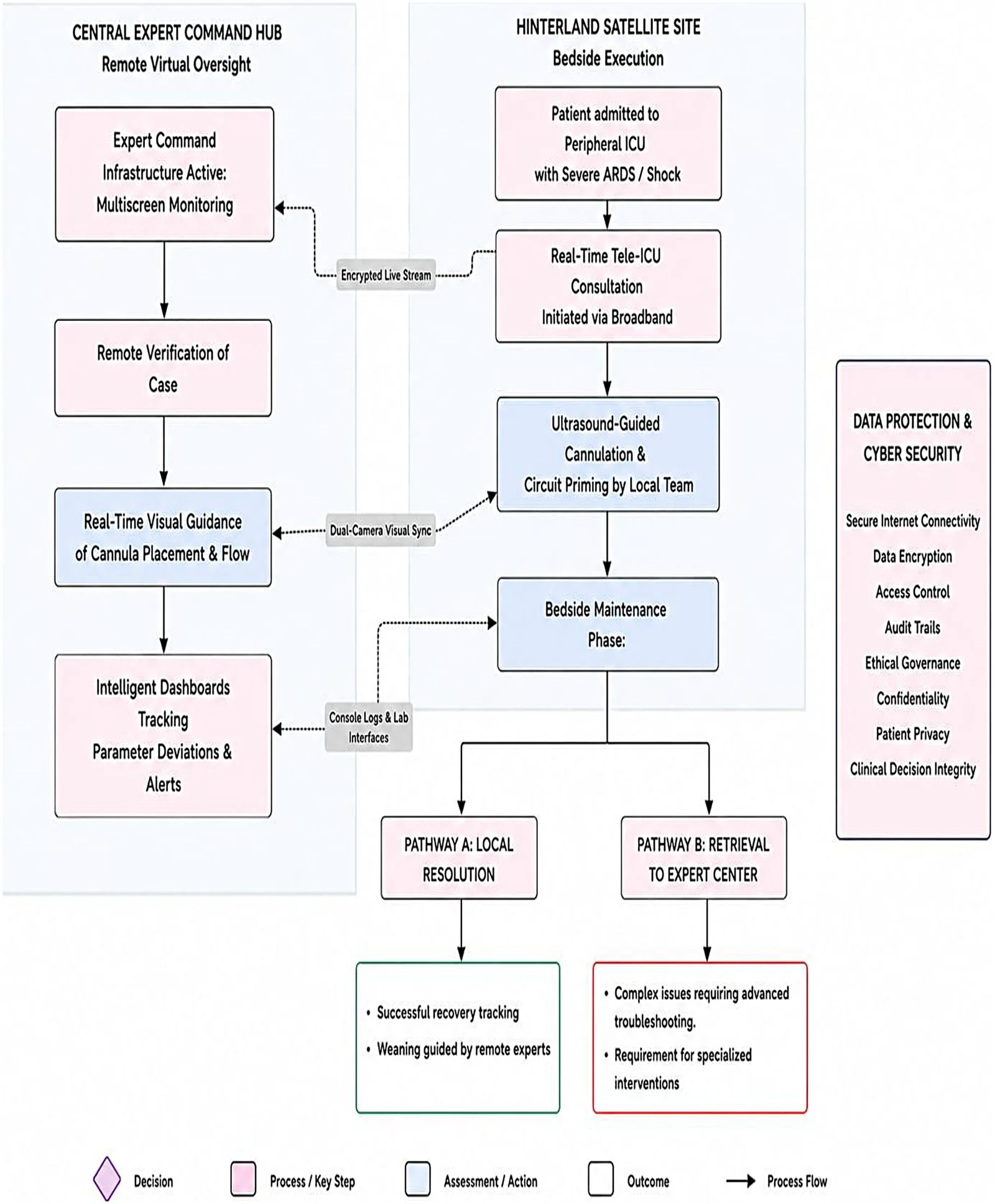
Flow chart of tele-ECMO. ECMO, extracorporeal membrane oxygenation.

**Table 1:** Summary of three models.

Type	Outreach team model	Hub-and-spoke model	Tele-ECMO model
Feature			
Core concept	An expert ECMO team travels to the peripheral hospital, initiates ECMO, and manages the patient locally for a limited period while supplementing local resources. (9)	ECMO is started at the spoke Centre by the hub team when deterioration is recognised, followed by transfer of the stabilised patient to the experienced hub (10).	A satellite Centre starts and manages ECMO locally under real-time digital supervision from a remote command centre by ECMO experts (38, 39).
Resources	Moderate to High:	High	Moderate:
	Depends on experience of peripheral hospital.	Need to arrange all resources for the time being.	
Primary location of care	At the peripheral Centre.	Initial stabilisation at the spoke, followed by transfer and care at hub.	At the satellite or peripheral Centre.
Source of Ability	Combination of outreach multidisciplinary team collaborating with local staff.	Retrieval team from the hub—takes care of cannulation, stabilisation, and transport to the hub (11, 49).	Centralised and is delivered virtually 24 × 7.
			But teams on the ground should have sound knowledge.
Major strengths	Brings ECMO closer to the patient and helps build long-term local ECMO capability through mentorship and training (4, 17, 33).	Enables initiation at the periphery, while ensuring definitive management in a high-volume centre—most practised (10, 23).	Extending expert oversight beyond geographic boundaries supports earlier initiation, improves standardisation, and reduces the risks of long-distance transfer (41, 42).
Major limitations	Resource-intensive, logistically demanding, strong local collaboration, governance, infection control, and contingency planning for complications or delayed transfer.	Highly dependent on early referrals, seamless coordination, cannulation, transport coordination, and management of high-risk transfer phases (31, 49, 50).	It depends on reliable connectivity, cybersecurity safeguards, trained bedside personnel, clear medico-legal governance, and sustained institutional investment (38, 40).
Transfer	Not an emergency.	Transfer is the crux of the model and usually follows after ECMO initiation and stabilisation.	Transfer is selective and coordinated remotely when advanced interventions such as surgery or other organ support are needed (39).
	Whenever required.		
Level of expertise	Low to moderate.	Moderate.	High.
Scalability	Moderate. Expansion depends mainly on the availability and mobility of experienced outreach teams.	High within an organised regional network, where one hub can support multiple spoke centres.	Potentially high. A well-established command centre can remotely support multiple appropriately trained satellite centres.
Technology dependence	Low to moderate.	Moderate.	Very high.

ECMO, extracorporeal membrane oxygenation.

## Types of ECMO

### ECMO encompasses various commonly used configurations

- **Veno-venous (VV) ECMO** supports severe respiratory failure without cardiac compromise (15).
- **Veno-arterial (VA) ECMO** supports both respiratory and circulatory failure, commonly used in cardiogenic shock (16).
- **Extracorporeal cardiopulmonary resuscitation (ECPR),** the most technically demanding ECMO application, is applied in cases of refractory cardiac arrest (17).

A major consideration in hinterland institutions is the type of ECMO initiated. For an incipient programme, Veno-venous (VV) ECMO is generally safer and more feasible because it avoids arterial cannulation and its attendant complications. Veno-arterial (VA) ECMO requires greater procedural skill, advanced haemodynamic monitoring, and immediate access to vascular or cardiothoracic surgical backup (16). Centres attempting VA-ECMO without adequate infrastructure risk serious complications that may outweigh potential benefits (6, 18).

### Common considerations across all models

Several foundational elements are common to ECMO initiation and maintenance, irrespective of the delivery model.

Early identification, consideration, and initiation of ECMO per ELSO guidelines, either via referral or with the help of an expert team, are extremely crucial. Evidence demonstrates that initiating extracorporeal membrane oxygenation (ECMO) within an optimal clinical window prior to the onset of irreversible organ dysfunction significantly improves patient outcomes (15, 19). To achieve this timely intervention, healthcare facilities must maintain the essential logistics outlined in (Supplementary Table S2).

The necessary infrastructure on the ground for initiation comprises dedicated ICU space, a dependable oxygen source, a power supply, ECMO consoles and circuits, a wide range of cannulas, ultrasonography, anticoagulation monitoring systems, and a blood bank are a few important items in the extensive checklist (6, 16, 20). Effective delivery of ECMO therapy demands a multidisciplinary team comprising ECMO physicians, intensivists, perfusionists, trained nursing staff, respiratory therapists, and vascular or cardiothoracic surgeons for backup.

Post-initiation of ECMO, irrespective of the type and place of maintenance, continuous monitoring of haemodynamics, ECMO monitors, circuit parameters, and coagulation status, along with routine parameters, is critical for patient recovery (20, 21). The ECMO programme must also integrate other organ support modalities, such as renal replacement therapy for acute kidney injury, which is required in the majority of ECMO patients, making it an indispensable adjunct (22). For cardiogenic shock, availability of an intra-aortic balloon pump at the centre can complement the VA-ECMO subset and enhance recovery (23). Additionally, the availability of other specialists is always an advantage. The availability of all resources in hinterland situations and timely interventions is of paramount importance.

During maintenance, complications requiring component replacement, troubleshooting, or emergency circuit exchange are among the most demanding aspects of ECMO care. Patel B et al. showed that rapid recognition and coordinated management of oxygenator failure, cannula malposition, distal ischaemia, and differential hypoxia are essential to prevent deterioration (24). The ability to safely perform emergency circuit exchanges is considered a key marker of programme maturity (25). Some severe complications may necessitate escalation to advanced interventions such as ventricular-assist devices or transplant evaluation, while limitations in peripheral centre expertise, resources, or family preference may prompt transfer to a higher centre, raising important ethical, clinical, logistical, and financial considerations (20, 26). Supplementary Table S3 shows the common indications where patients were transferred to expert centres from the hinterland.

Case volume is an important factor when establishing an ECMO programme. In the largest available registry analysis, adults managed at centres performing more than 30 ECMO cases per year had significantly lower risk-adjusted mortality than those treated at centres performing fewer than six cases (adjusted odds ratio 0.61) (18). More recent registry data suggest that this volume–outcome relationship persists for veno-arterial ECMO despite overall improvements in outcomes (27), a finding with direct practical relevance for peripheral centres. A centre unable to maintain adequate case volume, sustain 24 h surgical, perfusion, and vascular support, and conduct regular simulation-based training and competency assessment of its staff (28) may not be well placed to run an independent ECMO programme. Outreach or tele-ECMO models offer such centres an alternative, allowing them to draw on the expertise and experience of an established high-volume centre (6, 7).

Ethical governance is of paramount importance in this setting, requiring transparent, informed consent processes that clearly outline the objectives of ECMO, its associated risks, anticipated duration, withdrawal criteria, and financial implications (29). Ethics committees play a central role in guiding decisions about the continuation or withdrawal of support, particularly when clinical progress is poor or resources are limited (30). Comprehensive documentation and multidisciplinary involvement provide medicolegal clarity and protect the interests of patients, clinicians, and institutions, and institutions must ensure this process remains consistent with local legal frameworks throughout.

Infection control is a further critical concern in ECMO maintenance. Peripheral centres should uphold infection control standards on par with those of tertiary intensive care units, encompassing rigorous hand hygiene, meticulous line care, antimicrobial stewardship, and environmental sanitation (31). Engagement with the ELSO registry allows peripheral programmes to benchmark their outcomes, pursue quality improvement, and remain integrated within global data networks rather than operating in isolation (32).

Finally, programme quality demands ongoing review and cannot be secured through initial training alone. Morbidity and mortality meetings that involve the full multidisciplinary team and adhere to defined case-selection criteria are valuable not only for discussing individual cases but also for identifying system-level improvements (33). Participation in the ELSO registry (32) also allows a hinterland programme to compare its case-mix-adjusted outcomes with international benchmarks. More broadly, clinical registries across specialities have been associated with improvements in care processes and outcomes (34). Similarly, initial training alone may not be sufficient (35). Technical and cognitive skills required for uncommon but high-risk events, such as an emergency circuit exchange, should therefore be reassessed regularly through simulation, preferably annually (28).

Financial sustainability remains a significant challenge given the resource-intensive nature of ECMO, which is driven by equipment costs, specialised personnel, extended ICU stays, and ancillary interventions. Peripheral programmes may improve their viability through strategic staffing models, such as nurse-led or respiratory therapist-led ECMO under the supervision of perfusionists and physicians, as described by Odish et al. (36). Regional collaboration can also help consolidate resources and reduce the need for costly patient transfers (37). Equally important, though often overlooked, is the psychological well-being of staff: Passariello et al. highlight the necessity of structured psychological support to address burnout common in high-stress ECMO environments (38). Cost data specifically related to hinterland ECMO programmes remain limited. Available evidence, however, shows that ECMO care carries a substantial financial burden. In a large US cohort, the adjusted mean cost of an ECMO-associated hospital stay was USD 36,484, considerably higher than that of a comparable admission without ECMO (39). Consumables such as circuits, oxygenators, and cannulas contribute to this expense, but staffing is often a larger and less flexible component because ECMO requires dedicated nursing, ECMO specialists, and perfusionist support (40). Reimbursement structures vary considerably across healthcare systems and often fail to cover the full cost of ECMO, particularly in LMIC settings, where insurance coverage is limited and out-of-pocket costs remain common. Data on the cost-effectiveness of ECMO in peripheral and resource-limited settings remain scarce, and this area warrants further study.

### The ECMO outreach team

Case 1: A 50-year-old woman with uncontrolled diabetes, massive myocardial infarction, and refractory cardiogenic shock presented to the emergency department of a rural peripheral hospital. She underwent thrombolysis and was admitted to the ICU for observation. However, despite aggressive medical management, including vasopressors, inotropes, and eventually, mechanical ventilation, and initial stabilisation in the ICU, her condition continued to deteriorate. Echocardiography demonstrated severely compromised left ventricular systolic function, and her metabolic profile indicated worsening acidosis. Although ECMO was discussed as a salvage option and the family consented, they declined transfer to a tertiary ECMO-capable centre in a metropolitan city due to concerns about higher costs, leaving clinicians at a difficult crossroads.

This scenario is emblematic of a wider challenge: How should peripheral hospitals respond when ECMO is indicated, but system-level limitations prevent its deployment? Globally, access to ECMO remains restricted (1). Even in high-income nations, as suggested by Gottula et al. in a community survey in the United States, only about two-thirds of the population lives within reasonable proximity to an ECMO centre, which means the situation in developing regions with vast geographical areas is significantly more constrained (3). Nonetheless, emerging data challenge the assumption that ECMO must be confined to large academic centres. Peripheral hospitals, when provided with robust protocols, adequate training, and appropriate clinical governance, have reported survival rates comparable to those of major tertiary institutions during medical emergencies (4). Mobile ECMO programmes by Broman et al. and Sams et al. demonstrated that interfacility transport can be conducted with acceptable safety profiles (41, 42). However, despite this encouraging evidence, the structural obstacles faced by peripheral hospitals remain substantial.

A practical way to approach this conundrum would be to involve an outreach team fromexpert centre that is self-sufficient in initiating and managing ECMO for a few days at a peripheral centre. This would mean that the team would be fully equipped with ECMO-related resources, including a trained workforce to supplement resources at the peripheral centre (10–12). Responsibility for case selection, timing of consideration, and referral to the outreach team all lies with the peripheral institution. Such a model has not only helped to save lives in countries like India but has also enabled centres to start their own ECMO programme in the long run.

Supplementary Table S2 shows the logistics required for initiating ECMO, which are usually carried out by the outreach team, supplementing the necessary supplies on the ground at peripheral institutions. The outreach team during initiation and follow-up requires a support team, usually involving doctors from local institutions, and (Supplementary Tables S4 and S5) show the same minimal team composition.

For the local team to become self-sufficient, simulation-based training and mentorship from high-volume centres can help bridge initial gaps in expertise and understanding of the fundamentals of management (4, 20, 35). The model hinges on the outreach team's expertise in ECMO while utilising available resources at the peripheral hospital.

Standard ECMO care demands substantial institutional capacity, including dedicated space, specialised personnel, continuous laboratory monitoring, quality control, and established protocols for palliation or withdrawal of support, and peripheral centres must plan realistically for these resource demands before undertaking a programme. Figure 4 illustrates one such barrier, showing the limited ICU space characteristic of many remote healthcare settings. Outreach programmes should accordingly have a clear plan in place to manage these local constraints when ECMO is initiated and continued at a peripheral centre. Such endeavours should also define endpoints at which transfer to a tertiary or quaternary centre can be materialised (20, 26), consistent with the transfer indications already outlined (Supplementary Table S3). Awareness of legal considerations, informed consent, and transparent documentation are non-negotiable. Figure 1 shows a flow chart of the outreach programme.

**Figure 4:**
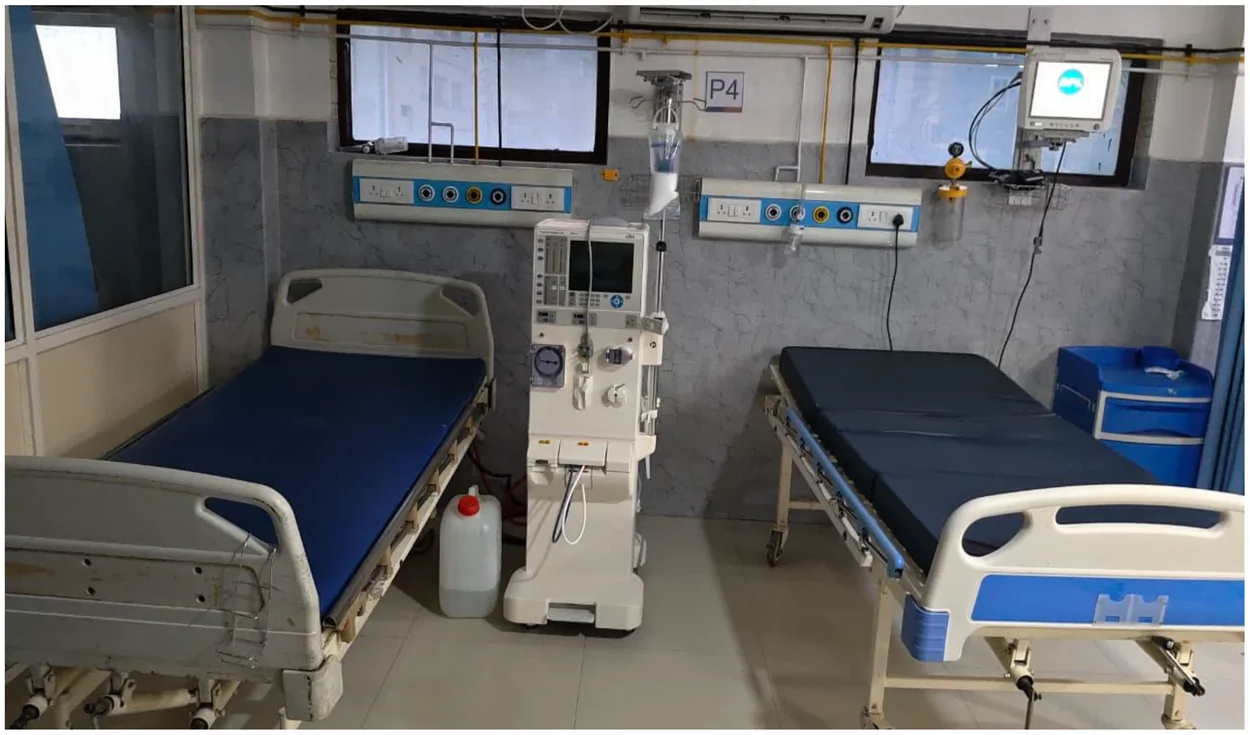
ICU AT hinterland institute—note space constraints.

The case of this patient with cardiogenic shock highlights a pervasive issue: the gap between technological capabilities and geographical access. ECMO has become central to advanced critical care, yet where a patient lives still determines whether they can receive it. The evidence reviewed here shows that ECMO can be delivered safely in peripheral hospitals when the right conditions are in place. The real question, then, is no longer whether ECMO can reach the hinterland, but how health systems build the structures, training, and support networks needed to get it there.

### Hub-and-spoke ECMO pathways in peripheral centres challenges, realities, and structured solutions

A well-proven concept in ECMO delivery is the hub-and-spoke model. Here, the hub is an ECMO expert centre, and the spoke is a peripheral institution synonymous with the hinterland. In this system, ECMO is initiated by the hub team at the spoke, where deterioration is first recognised; patients are then transferred to an experienced hub for further management once they become stable (13). The operational success of this network depends on the early identification of refractory pathophysiology, rapid clinical consultation, and seamless hub-and-spoke coordination. Empirical evidence underscores the critical importance of a highly efficient referral timeline, demonstrating that delayed transitions negatively impact patient survival (15, 19).

A representative clinical scenario illustrates the ecosystem's complexity.

Case 2: A 35-year-old man with severe hypoxaemic respiratory failure, worsening over three days despite escalating ventilatory support, including prone positioning and invasive mechanical ventilation, presented to a competent peripheral centre with strong pulmonary and critical care expertise. The patient's PaO_2_/FiO_2_ ratio remained at 100 despite rescue measures. The spoke centre recognises the trajectory toward refractory respiratory failure and contacts a high-volume ECMO hub for on-site initiation, followed by transfer after stabilisation.

This raises a crucial question: What constitutes a structured, safe, and reproducible plan of action in such circumstances? Unlike an outreach team, which is equipped to take care of ECMO patients in peripheral centres, the hub-and-spoke model offers its advantages and challenges (26, 43). This is the most practised model across the globe (12, 13, 26, 44) and is also demonstrated by the HandS (13) model in neonates. Figure 2 shows the ECMO hub-and-spoke flow chart.

Case selection at the spoke is one of the highest-stakes decisions in this model. Choosing between veno-venous (VV) and veno-arterial (VA) support is not a matter of ticking boxes—it means anticipating where the patient's physiology is heading, not just where it stands at the moment of referral. ELSO's guidelines lay out the framework for this decision (6), but applying them well still comes down to clinical judgement at the bedside.

“Appropriate patient” is a phrase that gets used a great deal and explained too little. Age is a good example: it should not be treated as a fixed cut-off. A large meta-regression found that mortality in VV-ECMO patients with ARDS—both COVID-19 and non-COVID-19—rose steadily with age, without any clear threshold beyond which the numbers turned sharply worse (45). Age rarely works alone, though. Among patients who were declined ECMO during the COVID-19 pandemic, advanced age, prolonged ventilation before cannulation, and multiorgan failure each independently predicted worse outcomes, with odds ratios of 4.4, 3.5, and 10.6, respectively (46). Severe irreversible neurological injury, profound frailty, and non-recoverable underlying disease should also be considered during assessment. At the same time, an international qualitative study found considerable variation in ECMO selection decisions between clinicians and institutions, influenced not only by formal criteria but also by local practice and cognitive bias (47). For hinterland centres without on-site specialist support, this variation highlights the importance of having predefined patient-selection criteria agreed upon with the hub or referring centre, rather than making decisions entirely on a case-by-case basis.

As discussed in the common things above, initiation logistics (Supplementary Table S2), teams for initiation (Supplementary Table S4), support teams (Supplementary Table S5), and others should be prepared on the ground. Post-initiation at the spoke, patient stabilisation through troubleshooting and transfer to the hub become crucial steps in this model.

Transport from the spoke to the hub is a complex extension of ECMO care. In-hospital transfer requires uninterrupted access through lifts and corridors, whereas road transport demands careful attention to battery backup, oxygen supply, power outlets, equipment fixation, and route planning (26). Supplementary Table S6 shows the logistics for keeping ready or carrying during air and ground transport. The ambulance team has to work at ICU-level vigilance throughout—watching for delays and obstacles along the route (Figure 5) and staying ready for an emergency stop if the patient deteriorates. Longer transfers bring their own demands: an escort or backup vehicle, rotating staff to avoid fatigue, and active measures to keep the patient warm. Pham et al. demonstrated in Vietnam that ECMO transport can be performed safely even in low-resource settings (12).

**Figure 5:**
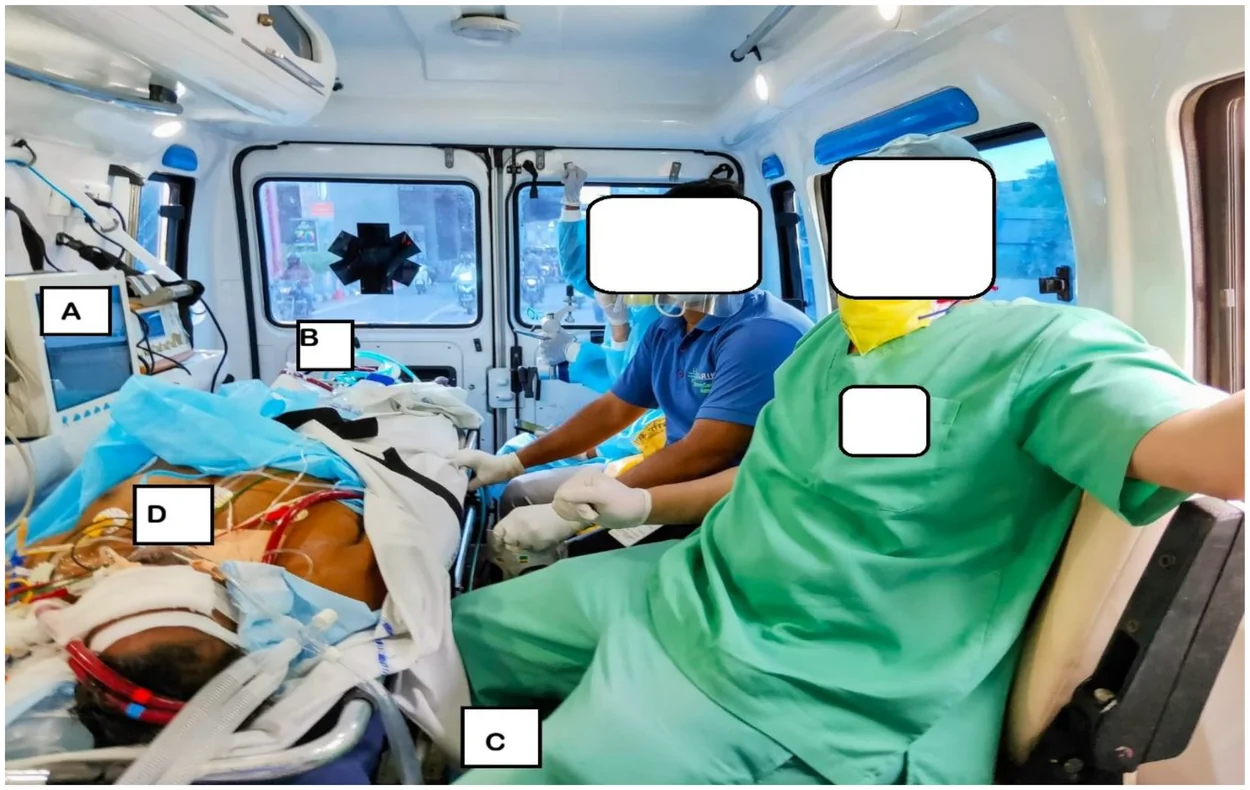
Image showing transport of ECMO patient via road ambulance. **(A)** monitor, **(B)** ECMO console between patient legs. **(C)** Constrained leg space, **(D)** patient with circuit. ECMO, extracorporeal membrane oxygenation.

Air transport adds a different set of problems. Cabin space is limited (Figures 6, 7), cabin pressure changes affect physiology, costs are high, payload is restricted, and the patient may need to be moved between vehicles more than once—all of which demand extensive preparation before the flight (26). Because of these combined risks, both road and air transport should be treated as high-risk phases of care in their own right. The best safeguard is a standardised safety checklist, an approach borne out by the Hub-and-Spoke (HandS) model and the protocols described by Mithu et al. (13, 48) (Supplementary Table S6).

**Figure 6:**
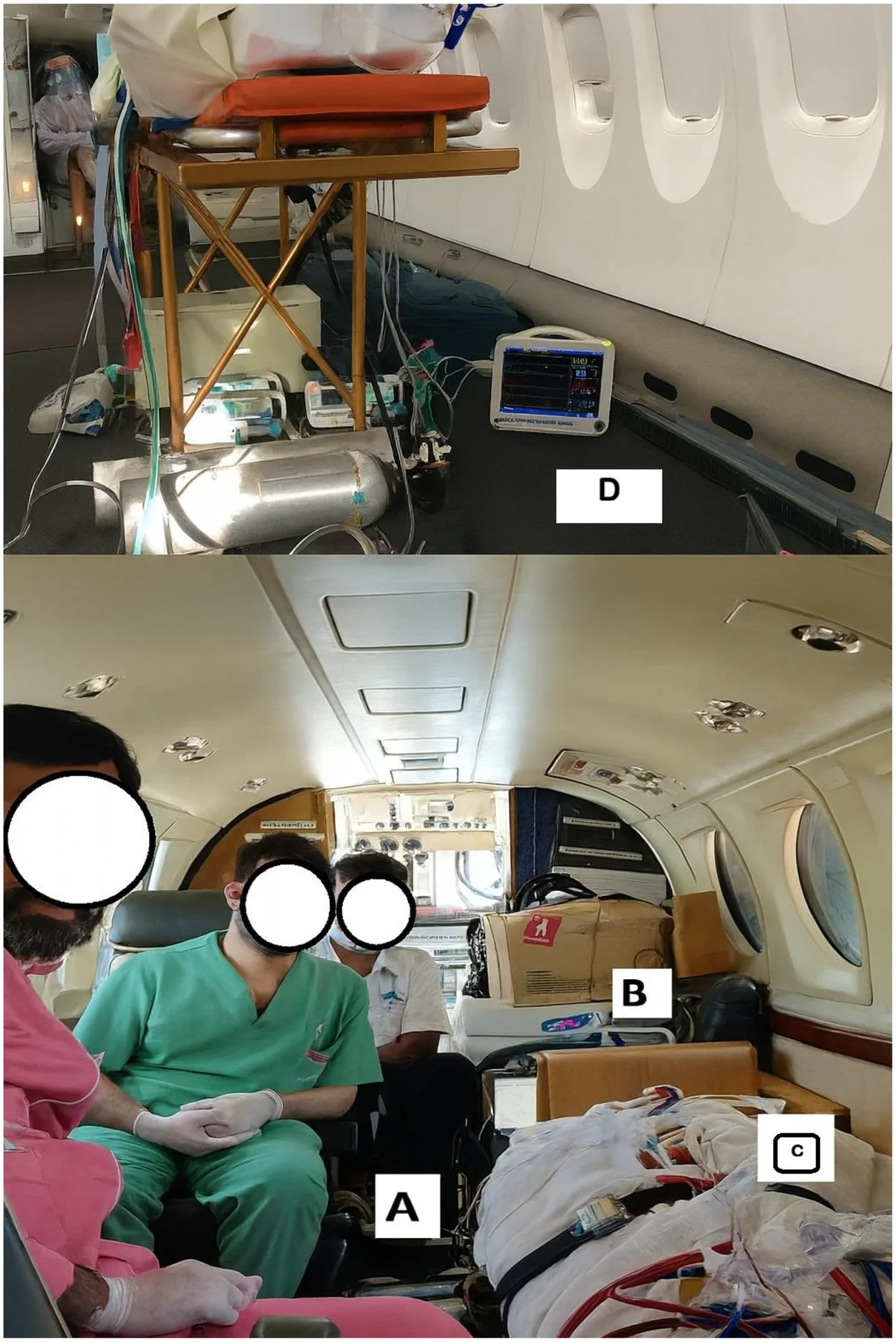
Image inside air ambulance. **(A)** free space available after loading of patient (working space), **(B)** infusion pump and emergency drugs, **(C)** patient with circuit, **(D)** inside ambulance showing monitor, cylinders, infusion pumps, and other instruments.

**Figure 7:**
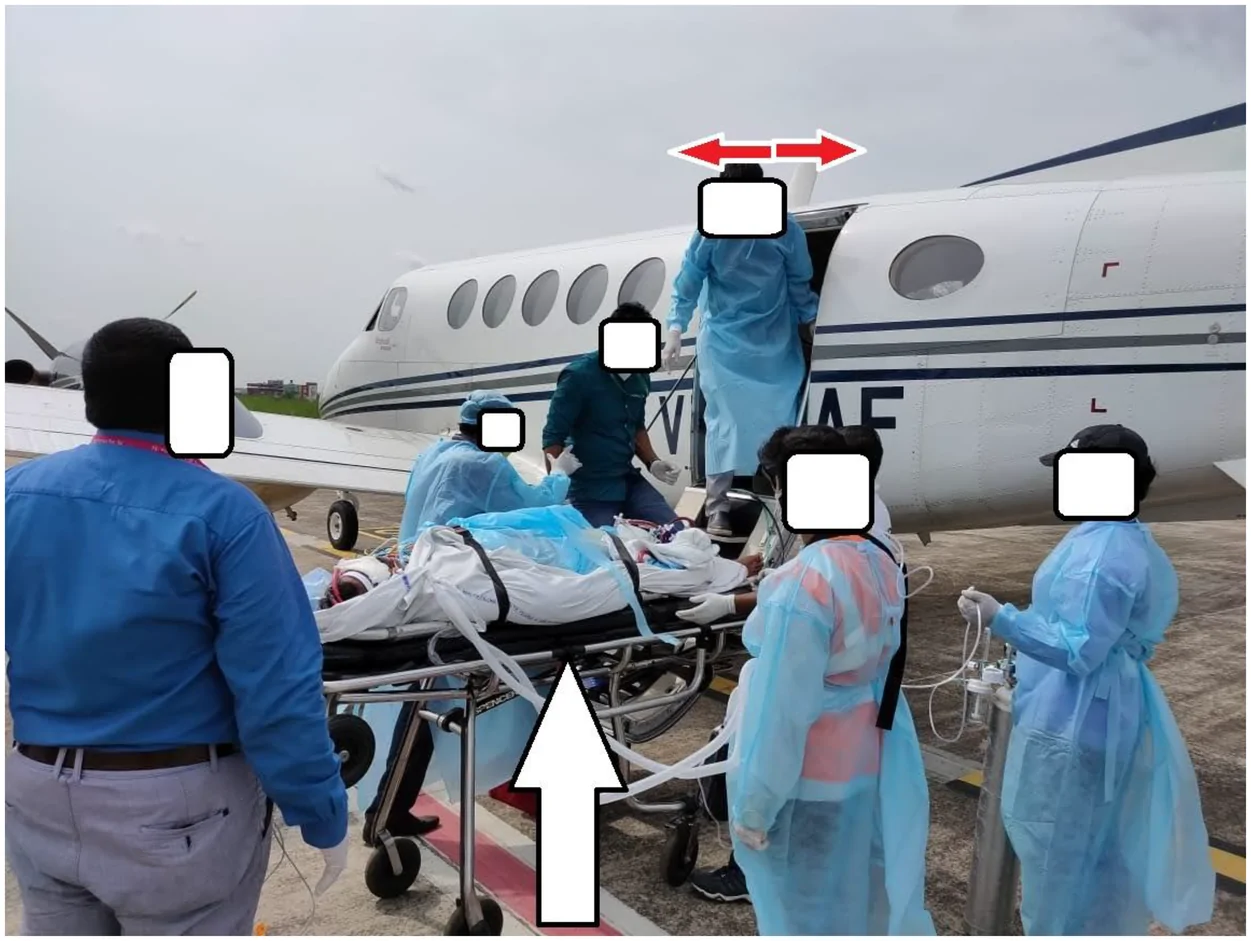
Transferring issues during patient transfer inside air ambulance, —white arrow indicating height to be lifted to load patient inside air ambulance, red arrows indicating narrow entrance of air ambulance.

Safe transport, on the ground or in the air, also comes down to the basics: maintaining correct alarm thresholds, securing cannulas and lines, monitoring anticoagulation, and staying in constant contact with the receiving ICU. Quick access to laboratory support, a transport checklist (Supplementary Table S6), and clear escalation protocols add another layer of safety. Just as important is starting the conversation early—about resource limits, the possibility that the transfer may not help, and who will handle the administrative and financial counselling so the family isn't left carrying that burden during an already difficult decision.

In summary, the successful implementation of a hub-and-spoke ECMO model in peripheral settings is achievable but requires disciplined coordination, anticipatory planning, and robust system-based practice. The key determinants of success—early referral, appropriate case selection, rigorous logistics, thorough counselling, and safe transport—must be consistently upheld (26). By strengthening communication channels, refining checklists, and establishing collaborative relationships between centres, peripheral hospitals can initiate ECMO promptly, while ensuring that patients ultimately receive care in high-volume expert environments. This integrated approach is essential for expanding ECMO access to underserved regions while maintaining safety, quality, and clinical excellence.

### Tele-ECMO: innovative model in modern critical care

In this model, the satellite centre is a peripheral centre, synonymous with the hinterland, but capable of initiating and maintaining ECMO independently under the command and supervision of a command centre. Both are connected live via the Internet in an era of technology such as tele-ICU models.

A 50-year-old man with severe ARDS secondary to influenza pneumonia had been supported on VV-ECMO for 10 days at a peripheral ECMO centre. During the course of ECMO, he developed progressive bleeding from the cannulation sites, oral, and tracheostomy sites, associated with thrombocytopenia. Despite conservative measures and periods of anticoagulation-free ECMO support, bleeding persisted. Simultaneously, the team observed increasing oxygenator clot burden with worsening post-membrane oxygenation on arterial blood gas analysis, indicating the need for oxygenator exchange.

The peripheral ECMO centre was functioning in collaboration with an expert centre through a tele-ICU model with continuous live guidance and remote specialist support. In such a scenario, is tele-supported ECMO management feasible and appropriate for handling complex complications? Additionally, how should informed consent and counselling be conducted with the patient's family?.

The ECMO Satellite–Command Centre model aims to bridge the service disparity by ensuring that expert oversight and real-time digital supervision from a command centre staffed by expert intensivists, perfusionists, and biomedical engineers, rather than geographic proximity, dictate patient outcomes (49). Through encrypted audiovisual links, live data streaming, and intelligent dashboards, the command hub provides continuous monitoring and decision-making support, transforming ECMO from an urban privilege to a widely accessible lifesaving intervention. Nick et al. presented a case report of a 39-year-old who was placed on ECMO and monitored for 72 h through an existing telecritical care network between two military centres (50).

At the heart of the model lies a clear conceptual division: the satellite centre serves as the physical site of ECMO initiation and bedside care, staffed by appropriately trained personnel, while the command centre functions as the supervisory hub that guides decision-making, maintains safety standards, and ensures uniformity of practice across multiple facilities. By decentralising ECMO away from a single central hub and enabling earlier initiation at the bedside, this model reduces delays, lowers the risks associated with late presentation, and puts advanced cardiopulmonary support within reach of centres that would otherwise be excluded from it (49–51). This isn't just a theoretical benefit—outcomes genuinely improve when ECMO starts earlier, under expert guidance, and follows processes the two centres have worked out together in advance, as set out in the flow chart in Figure 3.

At the command centre itself, multiscreen displays pull in real-time data from the ECMO console, ventilator, haemodynamic monitors, and laboratory systems simultaneously. A secure broadband link keeps audiovisual communication running continuously, so the remote team can actually see the patient, the console, and the room—not just read numbers off a screen. Colour-coded alerts flag any deviation in patient or machine parameters, borrowing the same logic as the tele-ICU model, so the team on the ground knows to act quickly when something changes (51–54).

A few clinical scenarios show how this plays out in practice. If a patient develops severe hypoxaemia, the satellite team can get the command centre on the line within minutes, send over real-time data, and get confirmation of ECMO eligibility almost immediately (49–51). From there, the remote experts guide the bedside team through ultrasound-guided vascular access, circuit priming, initiation of anticoagulation, and cannula placement, watching the entire procedure unfold via a dual-camera setup.

Things get more complicated when a patient needs to move to a tertiary facility for something the satellite centre can't provide—cardiac surgery, dialysis, or advanced diagnostics, for instance (Supplementary Table S3). Here, the command centre takes the lead: assessing the patient, preparing for the move, monitoring throughout the transit, and ensuring the handover is properly structured (41). GPS and 4G/5G connectivity mean that any critical change in flow, pressure, or oxygenation during the journey triggers a real-time automated alert. Standardised transport protocols have been shown in real-world studies to meaningfully reduce complications—further evidence for keeping oversight centralised (50–52).

As with the other two models, documentation and ethical governance matter just as much here. End-of-life decisions and other ethical questions should follow existing recommendations, adapted to this setting (29, 55). All data is transmitted over encrypted channels with strict access controls, and the use of any audiovisual material for teaching or documentation requires the patient's or family's explicit consent (53, 54). None of this holds together without ongoing training, though. Over time, satellite centres that engage in remote case reviews, simulation-based training, and structured competency assessments become genuine hubs of learning in their own right, not just delivery points (35, 56).

None of this is without its problems, though. Beyond the infrastructure issues already discussed, satellite centres need genuinely reliable internet connectivity—not just adequate on a good day (49, 51, 52). Hacking, software failures, and data loss are real threats that don't go away with good intentions. Communication lapses are just as dangerous in a quieter way: a documentation error or a mismatch in terminology between the two teams can translate directly into clinical risk. And none of this comes cheap—sustaining the system over the long term requires institutional and government backing (29, 55). To keep these risks in check, both the satellite and command centres should stay aligned with existing guidelines on AI use and telemedicine (53, 54).

Despite these limitations, the benefits of the tele-ECMO model remain profound. Tele-guided initiation reduces hypoxia-related injuries, whereas local management avoids risky long-distance transfers. Families experience greater reassurance through transparent monitoring, and healthcare systems gain access to continuous data streams that support quality audits, research, and policy development, in line with the established tele-ICU model and the guidelines issued by the country's respective authority (53, 54). Ultimately, the model transforms ECMO from a complex, centralised service into a scalable and equitable arm of modern critical care.

### ECMO in hinterland: future directions

Artificial intelligence may take this model further still, giving hinterland ECMO centres another route to strengthen their programmes as they mature.

Tele-ECMO, as it stands today, rests on solid ground: national critical care frameworks, telemedicine practice guidelines, and standardised certifications are already in place (53, 54). That gives remote consultation and the delivery of advanced organ support across different geographies a structured foundation to build on—nothing speculative about it.

In contrast, emerging AI-based advancements and predictive algorithms, such as the ECMOPAL model, represent a prospective frontier for enhancing patient selection and anticipating adverse events (57, 58). However, these technologies remain speculative due to significant data heterogeneity and a current lack of standardised ethical frameworks (59, 60), necessitating further validation before they can be integrated into routine clinical management.

### Critical analysis

Three models discussed as pathways for ECMO initiation and maintenance in hinterland scenarios each have their own challenges, limitations, and obstacles to overcome when establishing an ECMO programme. The most common model is the hub-and-spoke model, practised worldwide, and evidence supports safety during initiation and transport of ECMO patients (41, 61–63). Yang et al, Alexander et al., and Yuliya et al. demonstrated that ECMO transport in the hub-and-spoke model is not associated with significant complications or mortality (14, 61, 62). Survival rate in this model is compared with that of a high-volume centre, as the patient is transported back to the hub (14, 61, 62). Table 1 provides a direct comparison of the three models in terms of their core concept, resource requirements, primary location of care, major strengths, limitations, and transfer logistics. The following paragraphs discuss these differences in more detail.

The outreach programme faces major hurdles in case selection and the timing of the expert team's involvement. At the same time, ground preparation, team readiness and training of the ground team, and ECMO maintenance, in coordination with the expert team, also add to the list. The training burden is high here, and maintaining the ground team's skill set is the crux. Evidence supporting the outreach programme is sparse and comes from Dr Oza and others (10, 11), who notes that a few centres eventually developed their own ECMO programmes.

The teleECMO model: the major hurdle is selecting a centre that has already established a teleICU model with all required infrastructure. Another hurdle will be having a trained and confident ECMO team on the ground to initiate and maintain ECMO under the digital supervision of an expert team. Guidelines for operation, monitoring methods, digital connectivity, and remote expert team intervention are extrapolated from existing guidelines for teleICU and telemedicine (53, 54). Actual evidence supporting this model comes from experience during COVID times (49–51).

A direct comparison of these three models is difficult in view of the lack of evidence and a clear-cut comparison of parameters such as mortality, costs, and other factors.

### Limitations of this review

This review has several limitations. The literature search was restricted to PubMed and to a defined time period. Therefore, relevant studies indexed in Embase, Scopus, regional databases, or grey literature may not have been identified. This may also mean English-language studies indexed in PubMed are over-represented relative to the wider literature.

Most of what we know about these three delivery models comes from observational studies, registry data, case series, and expert opinion—not randomised trials. No study to date has directly compared outreach, hub-and-spoke, and tele-ECMO with respect to mortality, complications, or cost. The comparison offered in this review should be read as hypothesis-generating rather than the final word, and Table 1 as a narrative synthesis rather than a pooled statistical analysis.

Finally, some of the practical recommendations and cost estimates here come from the authors' own experience in middle-income settings, and may not translate directly to every hinterland centre worldwide. These limitations are worth keeping in mind when weighing the findings.

Overall, while transferring a patient from a peripheral centre to an ECMO centre remains standard practice after ECMO initiation, the possibility of ECMO maintenance in the hinterland presents its own set of opportunities and challenges. The ECMO outreach model enables ECMO in the hinterland with an expert team; it also helps handhold and build newer teams at the peripheral centre that can eventually manage ECMO referrals. The hub-and-spoke model relies on transferring ECMO patients to the hub centre soon after initiation; however, it still requires careful coordination and timely referral of eligible patients. Tele-ECMO represents a pivotal evolution in contemporary medicine, in which medical care is increasingly shaped by digital interconnectivity. The use of 5G technology in conducting telediagnostics in critical care can gradually evolve in the future to perform remote cannulation for ECMO and initiating ECMO (64). Telemonitoring would enable bridging of geographical and technical gaps during ECMO in the short term, and appropriate patients can be transferred to a centre of excellence to meet specific needs in the future. In an era of technological advancement, “distance should never determine the destiny” of a dying patient who would benefit from lifesaving interventions.
