# Supplementary material for: ECMO in hinterland—feasibility and challenges (case-based narrative review)
Source: Front Med (Lausanne). 2026 Sep 28;13:1778714. doi: 10.3389/fmed.2026.1778714 (PMC13659891; doi:10.3389/fmed.2026.1778714)
Supplement: Supplementary file 1 [file Supplementaryfile1.pdf]

SUPPLEMENTARY MATERIAL – (TABLE 2-6)

Table–2: CHECKLIST OF EQUIPMENT FOR INITIATION

No.	***ITEM NAME***
1	Rota flow/machine of choice
2	Drive unit
3	Hand crank
4	Sensors
5	Oxygenator with circuit
6	Cannulation kit
7	Airway bag
8	Fluids - balanced and dextrose
9	Ventilator
10	Monitoring Gear
11	Syringe pumps
12	Suction apparatus
13	Emergency medicines
14	Blood products
15	Oxygen cylinders
16	Bains circuit
17	Sterile gowns
18	USG machine
19	ACT machine
20	Scoop
21	AED
22	Manpower

USG – Ultrasonography, ACT – Activated clotting time, AED – Automated external defibrillator

Table-3: INDICATIONS TO SHIFT FROM HINTERLAND

INDICATIONS
Pregnant patients
Polytrauma patients
Patients requiring Cath lab interventions
Immunosuppressed patients
Patients requiring MCS devices
Transplant-listed patients
Neonatal and paediatric patients
Intractable complications arising during ECMO
Family request

MCS – Mechanical circulatory support, ECMO – Extracorporeal membrane oxygenation.

Table-4: ECMO INITIATION TEAM IN HINTERLAND AND THEIR ROLE

EXPERTS	*ROLE*
ECMO physician	Team lead/ cannulator / transport lead
Perfusionist	Circuit priming, machine functionality, and maintenance.
Vascular / CTVS surgeon	For cannulation/back up
Trained nursing staff - 1 or 2 in number	Logistics supervision. Checklist maintenance.<br><br>For help during cannulation

ECMO – Extracorporeal membrane oxygenation, CTVS – Cardiothoracic and vascular surgery.

Table-5: SUPPORT TEAM DURING ECMO INITIATION AT HINTERLAND AND THEIR ROLE

SUPPORT TEAM	*ROLE*
Critical care physician – 1 or 2	Volume Resuscitation, Haemodynamic Stabilisation, to lead things in case of emergency.
Nursing staff, Cath lab staff	Blood Products availability<br><br>Consents & Clearance
Vascular Sx & Perfusionist	As a back-up if available
Respiratory therapists/ EMT	Managing Emergency scenarios during cannulation

Sx – Surgeon, EMT- Emergency medical technician

Table -6: CHECKLIST FOR AMBULANCE (Ground & Air)

No.	***ITEM***	***GROUND***	***AIR***
1	Passport	No	Yes
2	Collect the latest fit-to-fly certificate from the hospital	No	Yes
3	Collect the Original Medical records and other documents	Yes	Yes
4	Family instructions shared	Yes	Yes
5	Valid ID card of patient and others	No	Yes
6	Emergency medicines	Yes	Yes
7	Consent	Yes	Yes
8	Transfer record form	Yes	Yes
9	Stethoscope & torch	Yes	Yes
10	Glucometer	Yes	Yes
11	Airway & Breathing bag	Yes	Yes
12	Circulation bag	Yes	Yes
13	ECMO equipment including hand crank	Yes	Yes
14	Infusion pumps	Yes	Yes
15	Ventilator	Yes	Yes
16	ET tube / NIV circuit	Yes	Yes
17	Suction apparatus	Yes	Yes
18	Cardiac Monitor & Defibrillator	Yes	Yes
19	Oxygen cylinders	Yes	Yes
20	Scoop and Recliner	Yes	Yes
21	Pillows / Bedsheets	Yes	Yes/no
22	Colloids	Yes	Yes
23	ABG machine with cartridges	Yes/ No	Yes /No
24	Ambulance checks on the departure side	Yes	Yes
25	Ambulance checks on the arrival side	Yes	Yes
26	Manifest check for air ambulance services team names - who accompanies the patient in the ambulance	No	Yes
27	Finances	Yes	Yes
28	Flight tickets	No	Yes
29	All equipment checked for sufficient battery/power back-up	Yes	Yes

ECMO – Extracorporeal membrane oxygenation, ET – Endotracheal, NIV – Non-invasive, ABG – Arterial blood gas
